# Characterization of quorum regulatory small RNAs in an emerging pathogen *Vibrio fluvialis* and their roles toward type VI secretion system VflT6SS2 modulation

**DOI:** 10.1080/22221751.2024.2396872

**Published:** 2024-08-28

**Authors:** Anran Zhang, Yue Xiao, Yu Han, Yuanming Huang, Biao Kan, Weili Liang

**Affiliations:** a National Key Laboratory of Intelligent Tracking and Forecasting for Infectious Diseases, National Institute for Communicable Disease Control and Prevention, Chinese Center for Disease Control and Prevention (China CDC), Beijing, People’s Republic of China; b Shanghai Pudong New Area Center for Disease Control and Prevention, Shanghai, People’s Republic of China

**Keywords:** Quorum regulatory small RNAs (Qrr sRNAs), type VI secretion system (T6SS), LuxO, HapR, *Vibrio fluvialis*

## Abstract

The type VI secretion system (T6SS) is essential for Gram-negative bacteria to antagonize a wide variety of prokaryotic and eukaryotic competitors and thus gain survival advantages. Two sets of T6SS have been found in *Vibrio fluvialis*, namely VflT6SS1 and VflT6SS2, among which VflT6SS2 is functionally expressed. The CqsA/LuxS-HapR quorum sensing (QS) system with CAI-1 and AI-2 as signal molecules can regulate VflT6SS2 by regulators LuxO and HapR, with LuxO repressing while HapR activating VflT6SS2. Quorum regulatory small RNAs (Qrr sRNAs) are Hfq-dependent *trans*-encoded sRNAs that control *Vibrio* quorum sensing. In *V. fluvialis*, Qrr sRNAs have not been characterized and their regulatory function is unknown. In this study, we first identified four Qrr sRNAs in *V. fluvialis* and demonstrated that these Qrr sRNAs are regulated by LuxO and involved in the modulation of VflT6SS2 function. On the one hand, Qrr sRNAs act on HapR, the activator of both the major and the auxiliary clusters of VflT6SS2, and then indirectly repress VflT6SS2. On the other hand, they directly repress VflT6SS2 by acting on *tssB*2 and *tssD*2_a, the first gene of the major cluster and the highly transcriptional one among the two units of the first auxiliary cluster, respectively. Our results give insights into the role of Qrr sRNAs in CAI-1/AI-2 based QS and VflT6SS2 modulation in *V*. *fluvialis* and further enhance understandings of the network between QS and T6SS regulation in *Vibrio* species.

## Introduction

After being isolated from patients with diarrhoea in 1970s, *Vibrio fluvialis* has emerged as an unusual enteric pathogen of increasing public health concern [1–3]. Like other *Vibrio* species, *V*. *fluvialis* can be widely found in aquatic environments, especially in seas, estuaries, and brackish waters [4]. People can become infected with *V*. *fluvialis* through the consumption of raw shellfish or contaminated seafood products [5]. As a foodborne pathogen, *V*. *fluvialis* is often associated with sporadic cases and outbreaks of diarrhoea and its clinical symptoms of gastroenteritis are very similar to those of *Vibrio cholerae* [6–8]. *V*. *fluvialis* has also been reported to cause extraintestinal infections such as haemorrhagic cellulites and cerebritis, peritonitis, acute otitis, biliary tract infection, bacteraemia and even ocular infections [4]. Although many putative virulence factors have been detected, the pathogenic mechanisms of *V*. *fluvialis* are largely unknown. In recent years, the emergence of multidrug-resistant isolates of *V*. *fluvialis* raised its public health significance [9].

Quorum sensing (QS) is a cell–cell communication process that bacteria use to monitor changes and coordinate behaviour in response to cell-population density. QS relies on the production, release, detection and population-wide response to extracellular signal molecules called autoinducers [10]. The quorum regulatory small RNAs (Qrr sRNAs) are Hfq-dependent *trans*-encoded sRNAs function at the centre of QS pathways in *Vibrios* [11]. Qrr sRNAs can regulate multiple mRNA targets of the QS regulatory components and exert both positive and negative effect [12]. In *Vibrio harveyi*, under low cell density (LCD) conditions, the phosphorylated LuxO (LuxO∼P) promotes the transcription of Qrr sRNAs, which stimulate the translation of QS regulator AphA and inhibit the translation of regulator LuxR. Under high cell density (HCD) conditions, the dephosphorylation of LuxO represses the transcription of Qrr sRNAs, leading to the translation of LuxR [13]. Qrr sRNAs are also found to post-transcriptionally regulate gene expression outside of the QS circuit [14].

Type VI secretion system (T6SS) is a contact-dependent bacterial weapon through which effectors can be delivered to neighbouring prokaryotic and eukaryotic competitors, exerting the effect of killing target cells [15]. T6SS is of great significance for pathogenicity and environmental adaptability of bacteria. T6SS was first identified in *V*. *cholerae* and is widely distributed in Gram-negative bacteria [16,17]. In *V*. *cholerae*, the activity of T6SS is regulated by QS through Qrr sRNAs [18]. At LCD, the LuxO∼P activates the expression of four Qrr sRNAs known as Qrr1–4, which bind to and destabilize the 5´ UTR of the mRNA transcripts of the major gene cluster of the T6SS and HapR, thus repressing T6SS. At HCD, LuxO is unphosphorylated and transcription of Qrr1–4 is inactive, permitting the translation of the major gene cluster of T6SS and HapR, so activating T6SS. Therefore, in *V*. *cholerae*, Qrr sRNAs function between LuxO and HapR in QS cascade, regulating T6SS activity.

In *V*. *fluvialis*, two QS systems have been identified [19]. One is acyl-homoserine lactone (AHL) based VfqI-VfqR system and the other is cholerae autoinducer-1 (CAI-1) and autoinducer-2 (AI-2) based CqsA/LuxS-HapR system [19]. Meanwhile, two sets of T6SS gene clusters, namely VflT6SS1 and VflT6SS2, have been identified [20]. VflT6SS2, consisting of one major gene cluster and three *hcp*-*vgrG* auxiliary gene clusters, is functionally expressed and mediates bacterial killing effect [20]. Both CqsA/LuxS-HapR and VfqI-VfqR QS systems can modulate VflT6SS2, with the former playing a major regulatory role [21,22]. HapR can physically bind to the promoter regions of both the major and auxiliary clusters of VflT6SS2, directly activating VflT6SS2. Whether Qrr sRNAs exist in *V*. *fluvialis* and play roles between LuxO and HapR to modulate VflT6SS2, like the mode existed in *V*. *cholerae*, is unknown.

In this study, the Qrr sRNAs in *V*. *fluvialis* were characterized and their function in VflT6SS2 modulation was explored. Four Qrr sRNAs were identified and they are regulated by LuxO. The deletion of sRNA chaperone Hfq increases the expression and secretion of the Hcp protein of VflT6SS2 and the VflT6SS2-mediated bactericidal ability of *V*. *fluvialis*. Qrr4, the most highly expressed Qrr sRNA, can directly regulate the expressions of *hapR*, *tssB*2 and *tssD*2_a by targeting to the 5´ UTRs of their mRNAs. Together, our results provided insights into the Qrr sRNAs in *V*. *fluvialis* and their function between the regulation of QS and VflT6SS2. Importantly, the functional model of Qrr sRNAs toward T6SS modulation in *V*. *fluvialis* is different from that in *V*. *cholerae*, indicating diverse mechanisms of Qrr sRNAs used by pathogenic *Vibrios* for infection and survival.

## Materials and methods

### Bacterial strains, plasmids and media

The bacterial strains and plasmids used in the present study are listed in Tables S1 and S2. The wild-type (WT) strain of *V*. *fluvialis* 85003 and its derivative mutant strains were grown in Luria–Bertani (LB) medium or agar plates containing 1% (for general growth) or 2% NaCl (for VflT6SS2 activation) at 30℃. *Escherichia coli* strains (DH5α*λpir*, SM10*λpir*, BL21(DE3) and MG1655) were cultured at LB medium or agar plates containing 1% NaCl at 37℃. These *E*. *coli* strains were used for the following purposes: DH5α *λpir*, transformation; SM10 *λpir*, conjugation; BL21(DE3), expression of exogenous genes; MG1655, prey in bacterial killing assay. Antibiotics were added as needed at the following final concentrations: ampicillin (Amp), 100 μg/mL; streptomycin (Sm), 100 μg/mL; rifampicin (Rfp), 50 μg/mL; chloramphenicol (Cm), 10 μg/mL for *E*. *coli* and 3 μg/mL for *V*. *fluvialis*; kanamycin (Km), 50 μg/mL. Isopropyl β-D-Thiogalactoside (IPTG) was added for induction as needed at a final concentration of 0.01 mM.

### Construction of mutants and recombinant plasmids

*V*. *fluvialis* Δ*hfq* mutant strain was constructed by allelic exchange based on WT strain 85003. Briefly, the upstream and downstream DNA fragments flanking *hfq* open reading frame (ORF) were amplified using primer pairs *hfq*-F1-up-XhoI/*hfq*-F1-dn and *hfq*-F2-up/*hfq*-F2-dn-*Sma*I, respectively. The amplicons were then mixed and used as the templates for overlapping PCR using primer pair *hfq*-F1-up-XhoI/ *hfq*-F2-dn-*Sma*I to amplify the DNA fragment containing the *hfq* deletion. The resulting fragment was then cloned into suicide plasmid pWM91 to generate pWM-Δ*hfq*, which was subsequently transformed to SM10*λpir* and finally transferred to strain 85003 by conjugation. Exconjugants were selected on LB agar plates containing Amp and Sm, and the resistant clones were then streaked on NaCl-free LB agar plates containing 10% (w/v) sucrose for second selection. Sucrose-resistant clones were tested for Amp sensitivity and Sm resistance, before confirming the deletion of *hfq* by PCR and DNA sequencing.

*V*. *fluvialis luxO*D47E point mutation strain was constructed based on strain 85003 in the same manner of allelic exchange as above described, except that primer pairs *luxO*D47E-F1-F-*BamH*I/*luxO*D47E-F1-R and *luxO*D47E-F2-F/*luxO*D47E-F2-R-*Sac*I were used for the first PCR and primer pair *luxO*D47E-F1-F-*BamH*I/ *luxO*D47E-F2-R-*Sac*I was used for the second overlapping PCR. The introduction of D47E point mutation generates a *Xho*I restriction site, which is absent in wildtype strain and can be used for D47E confirmation.

*V*. *fluvialis* Δ*hfq luxO*D47E mutant strain was constructed by transferring the pWM-*luxO*D47E plasmid into Δ*hfq* strain through conjugation. The following steps for selection and confirmation were the same as above described.

To construct recombinant *lux* bioluminescence reporter plasmids with WT *qrr* promoter, the DNA fragment containing the promoter region of *qrr* was amplified with corresponding primer pair in Table S3 and ligated into the promoterless pBBR*lux* plasmid. The recombinant pBBR*lux*-based plasmids with mutant *qrr* promoter were constructed by Beijing Tsingke Biotech Co., Ltd. The synthetic DNA sequences were listed in Table S4. The resultant pQrr-*lux* plasmid was then transferred into SM10*λpir* and then mobilized into strain 85003, Δ*luxO* or *luxO*D47E by conjugation. The recombinant plasmids based on pBAD33 or pET-28a-EGFP-c vectors were constructed by Beijing Tsingke Biotech Co., Ltd. The synthetic DNA sequences were listed in Table S4. The corresponding recombinant plasmids was co-transformed into BL21 according to the following combination: pET-5′ UTR *hapR*-EGFP and *qrr*4-pBAD33; pET-5′ UTR *hapR*-EGFP and anti-*qrr*4-pBAD33; pET-5′ UTRm *hapR*-EGFP and *qrr*4-pBAD33; pET-5′ UTR *tssB*2-EGFP and pBAD33; pET-5′ UTR *tssB*2-EGFP and *qrr*4-pBAD33; pET-5′ UTR *tssB*2-EGFP and anti-*qrr*4-pBAD33; pET-5′ UTR *tssD*2_a-EGFP and pBAD33; pET-5′ UTR *tssD*2_a-EGFP and *qrr*4-pBAD33; pET-5′ UTR *tssD*2_a-EGFP and anti-*qrr*4-pBAD33.

### Bioinformatic analysis

The sequences of *qrr* homologues from representative *Vibrio* strains were obtained from the NCBI database under the following accession numbers: *V*. *cholerae* N16961 (GCF_000006745.1), *V*. *parahaemolyticus* RIMD 2210633 (GCF_000196095.1), *V*. *vulnificus* YJ016 (GCF_000009745.1), *V*. *harveyi* ATCC 33843 (GCF_000770115.1) and *V*. *fluvialis* ATCC 33809 (GCF_001558415.2). Sequence alignment of *qrr* genes was performed by Genedoc (version 2.7). UNAFold web server (RNA folding form, version 2.3, http://www.mfold.org/mfold/applications/rna-folding-form-v2.php) was used to predict RNA secondary structures [23]. RNAhybrid (https://bibiserv.cebitec.uni-bielefeld.de/rnahybrid) [24] was used to predict potential base-pairs between Qrr4 of *V*. *fluvialis* 85003 and its target mRNA.

### Luminescence activity assay

The 30℃ overnight culture of *V*. *fluvialis* WT strain or mutants containing pQrr1, 2, 3 or 4-*lux* recombinant plasmid was collected and then diluted (1:100) with fresh LB medium. 200 μL diluted culture was transferred into an opaque-wall 96-well microtiter plate (Corning, USA) and incubated at 30℃. Three biological replicates were set for each sample and three technical repeats were set for each replicate. The luminescence value (Lux, Integration time at 1000 ms) and the OD_600_ (Absorbance at 600 nm) value of each repeat were measured at corresponding time points. The relative luminescence intensity RLU (Lux/OD_600_) was calculated to reflect the promoter activity.

### Western blot analysis

The 30℃ overnight culture of *V*. *fluvialis* WT strain or mutants was collected and then diluted (1:100) with fresh LB medium. The diluted culture was then incubated overnight at 30℃ again, followed by the same procedure of dilution mentioned above. The twice-diluted culture was then incubated at 30℃ until the OD_600_ value reached 0.5–0.8 (logarithmic phase). Subsequently, the bacterial pellet and supernatant were collected by centrifugation at 4℃. The following treatment of bacterial pellet and supernatant was as previously described [20]. The protein concentration was measured by Pierce^TM^ BCA protein assay kit (Thermo Fisher Scientific, USA). For western blot, protein samples in loading buffer were denatured at 100℃ for 10 min and separated by SDS-PAGE. Protein bands were transferred to polyvinylidene difluoride (PVDF) membranes (Millipore, Billerica, MA, USA) and blocked overnight with TBS-Tween 20 containing 5% skim milk. Anti-Hcp [20] and anti-cyclic AMP receptor (Crp, *E*. *coli*) (BioLegend, USA) antibodies were used as the primary antibodies. The secondary antibodies used were horseradish peroxidase (HRP)-conjugated goat anti-rabbit and goat anti-mouse antibodies. Proteins were visualized by Enhanced Chemiluminescence system (TaKaRa, Japan) and photographed by ChemiDoc XRS + (Bio-Rad, USA). Three independent experiments were performed for each sample. ImageJ was used to analyze the gray scale of target proteins [25].

### Bacterial killing assay

This assay was performed as previously described [20]. Briefly, the *V*. *fluvialis* WT or mutant strains and *E*. *coli* MG1655 strain were cultured overnight on LB agar plates with corresponding antibodies. Then the predator strain (*V*. *fluvialis* WT or mutant) was mixed with the prey strain (*E*. *coli* MG1655) at a ratio of 9:1. A total of 10 µL mixture was spotted on filter membrane on LB agar plate and incubated at 30℃ for 5 h. The colony-forming units (CFUs) of the predator and the prey before (T0) and after (T5) the 5-h mixed incubation were measured by 10-fold serial dilutions and plate counting. The survival of prey (*E*. *coli* MG1655) at T5 and T0 was calculated to reflect the bactericidal ability of *V*. *fluvialis*. Three independent experiments were performed for each sample.

### RNA extraction and quantitative reverse transcription PCR (qRT-PCR)

*V*. *fluvialis* WT or mutant strains were cultured to reach early logarithmic phase (OD_600_ at 0.2) or later logarithmic phase (OD_600_ at 1.5). Total RNA extraction and cDNA synthesis were performed as previously described [26]. qRT-PCR was performed by CFX96 Touch Real-Time PCR Detection System (Bio-Rad, USA) using SYBR Premix Ex Taq (TaKaRa, Japan). Relative expression values (*R*) were calculated using the equation *R* = 2^−(ΔCq target−ΔCq reference)^, in which Cq is the fractional threshold cycle. *recA* was used as internal reference and unreversed-transcribed RNA as negative control. Primers used were listed in Table S3.

### GFP reporter assay

The *E*. *coli* BL21 strains containing pBAD33 and pET-28a-EGFP-c-based recombinant plasmids were incubated overnight at 37℃. Then the overnight culture was diluted (1:1000) with fresh LB medium and continually incubated to reach OD_600_ at 0.4∼0.6, before the addition of 0.01 mM IPTG and 0.4% L-Arabinose for induction (the addition of 0.01 mM IPTG but not 0.4% L-Arabinose was set as negative control). At every hour, 200 μL bacterial suspension was transferred to an opaque-wall 96-well microtiter plate to measure the fluorescence units (Excitation at 480 nm and Emission at 520 nm) and the OD_600_ (Absorbance at 600 nm) values. The relative fluorescence units RFU (fluorescence unit/OD_600_) were then calculated.

### Statistics

GraphPad Prism software, version 8, was used for statistical analysis. Two-way analysis of variance (ANOVA) was used for two-variable comparisons, while one-way ANOVA was used for one-variable comparisons. *P* values of <0.05 were considered to be significant.

## Results

### Characterization of Qrr sRNAs in V. fluvialis

Previous studies have found four Qrr sRNAs in *V*. *cholerae*, and LuxO∼P together with σ^54^ regulate the expression of these Qrr sRNAs [11]. Using the *qrr* genes in *V*. *cholerae* as queries, sequence alignment was performed to identify homologues in the genome of *V*. *fluvialis* strain 33809. Four putative *qrr* genes (*qrr*1, *qrr*2, *qrr*3 and *qrr*4) encoding four Qrr sRNAs (Qrr1, Qrr2, Qrr3 and Qrr4) were identified in *V*. *fluvialis* strain 33809, with the length of 112, 119, 120 and 121 bp, respectively.

**Figure 1.**
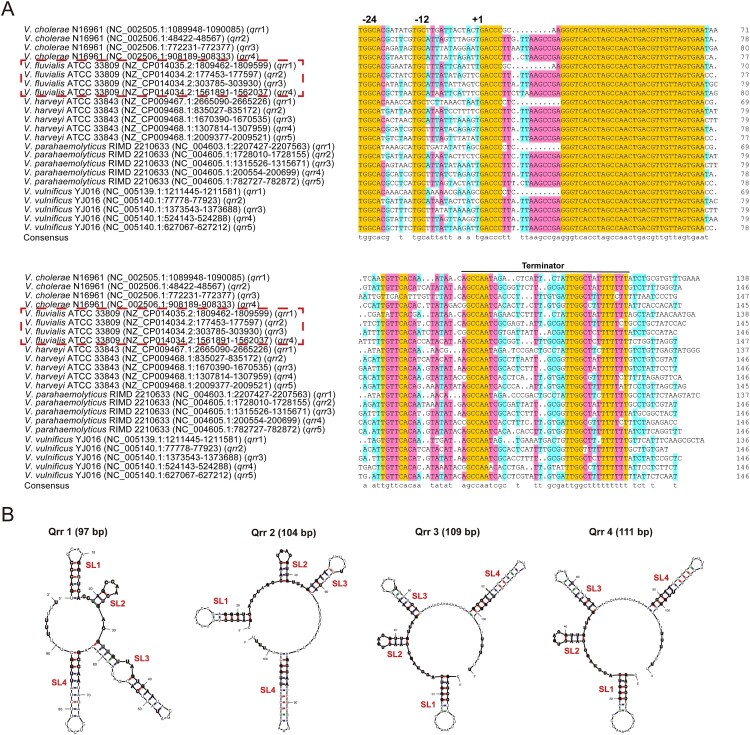
Sequence alignment of the *qrr* genes in five *Vibrio* species and the predicted secondary structures of four Qrr sRNAs in *V*. *fluvialis* 33809. (A) Sequence alignment of the *qrr* genes encoding Qrr sRNAs in five *Vibrio* species (*V*. *cholerae*, *V*. *fluvialis*, *V*. *harveyi*, *V*. *parahaemolyticus*, and *V*. *vulnificus*). The strain names are next to the corresponding species name. The NCBI accession number and the location information of each *qrr* genes are given in the brackets. For *V*. *cholerae* N16961 and *V*. *fluvialis* 33809, *qrr*1–4 are indicated, while for *V*. *harveyi* ATCC 33843, *V*. *parahaemolyticus* RIMD 2210633 and *V*. *vulnificus* YJ016, *qrr*1–5 are indicated. The putative σ^54^ binding site is marked as -12 and -24. The predicted transcription start site is labelled as +1. The predicted terminator is noted by the line over the sequences. The length of each aligned sequence is labelled on the right. Nucleotides in orange indicated 100% homology, while nucleotides in pink and light blue indicated ≥75% and ≥50% homology, respectively. The consensus sequence is indicated on the bottom. (B) Lowest-energy secondary-structural predictions for the four Qrr sRNAs identified in *V*. *fluvialis* 33809. The predicted stem-loops (SLs) are labelled. Nucleotides in the conserved regions in panel (A) are shown in bold.

We further aligned the promoter sequences upstream of the putative transcription start sites (TSSs) and the coding sequences of *qrr*1–4 in *V*. *fluvialis* strain 33809 and corresponding gene sequences in *V*. *cholerae* strain N16961, *V*. *parahaemolyticus* strain RIMD 2210633, *V*. *vulnificus* strain YJ016 and *V*. *harveyi* strain ATCC 33843 [11,27]. As shown in Figure 1A, *qrr*1–4 in *V*. *fluvialis* showed high homology with the *qrr* genes in other *Vibrio* species. All aligned sequences showed conserved -24 and -12 regions, indicating the activation by σ^54^. The predicted lowest free energy secondary structures of Qrr1–4 in *V*. *fluvialis* were showed in Figure 1B. Four regions forming predicted stem loops (SL1, SL2, SL3, and SL4) were identified for each Qrr sRNA. The predicted structures of Qrr2, Qrr3, and Qrr4 are similar.

### Luxo is essential for the promoter activity of **Qrr sRNAs in V. fluvialis**

**Figure 2.**
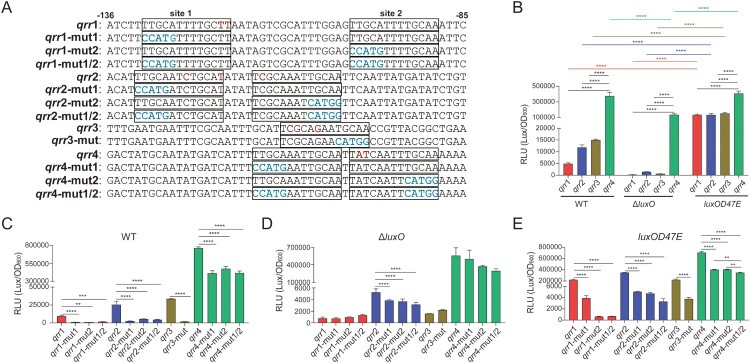
LuxO promotes the promoter activity of Qrr sRNAs in *V*. *fluvialis*. (A) Sequence alignment of the promoter regions of the four *qrr* genes in *V*. *fluvialis* 85003. The numbers above the sequences indicate the corresponding positions of nucleotides. The putative LuxO-binding sites are labelled by boxes. In each box, nucleotides different from the consensus sequence (5′-TTGCAW_3_TGCAA-3′) are marked by red. For each *qrr* gene, mutations were made in the conserved half-site of the palindromic sequence (5′-TTGCAW_3_TGCAA-3′) and shown in blue. For *qrr*1, *qrr*2 and *qrr*4, which contain two putative LuxO-binding sites, site 1 and 2 are labelled. Briefly, mut1 represents the mutation of site 1; mut2 represents the mutation of site 2; mu1/2 represents the simultaneous mutation of site 1 and 2. For *qrr*3, which contains only one putative LuxO-binding site, only *qrr*3-mut was made. (B) The promoter activity of each *qrr* gene in WT, Δ*luxO* and *luxO*D47E strains. The overnight cultures of WT, Δ*luxO* and *luxO*D47E strains containing pQrr1-*lux*, pQrr2-*lux*, pQrr3-*lux*, or pQrr4-*lux* were diluted (1:100) in LB medium and incubated at 30°C with shaking (200 rpm). The luminescence (Lux) was measured by transferring 200 µL aliquots of each culture into an opaque-wall 96-well microtiter plate. Relative luminescence activity (RLU) is calculated as Lux/OD_600_. The data represent the means ± standard error of mean (SEM) of three independent biological replicates. Two-way ANOVA with Tukey correction for multiple testing; asterisks represent a significant difference from WT as follows: ****, *P *< 0.0001. (C–E) The promoter activity of each *qrr* gene containing mutation in the putative LuxO-binding site in WT (C), Δ*luxO* (D), and *luxO*D47E (E) strains. The experimental procedure and the calculation of RLU are the same as above. The data represent the means ± standard error of mean (SEM) of three independent biological replicates. One-way ANOVA with Tukey correction for multiple testing; asterisks represent a significant difference from WT as follows: **, *P *< 0.01; ***, *P *< 0.001; ****, *P *< 0.0001.

In *V*. *cholerae* and *V*. *harveyi*, LuxO phosphorylation, together with σ^54^, can activate the expression of Qrr sRNAs at LCD [11]. To investigate whether the same regulatory mode exists in *V*. *fluvialis*, we firstly analyzed the upstream promoter region of *qrr* genes. As shown in Figure 2A, putative LuxO-binding sites (TTGCAW_3_TGCAA, W refers to A/T) [11] were identified for each *qrr* gene. For *qrr*1, two possible LuxO-binding sites were identified, located at -119 bp and -89 bp upstream of the predicted TSS, respectively; For *qrr*2, two possible LuxO-binding sites were identified, located at -120 bp and -103 bp upstream of the predicted TSS, respectively; For *qrr*3, only one possible LuxO-binding site was identified, located at -99 bp upstream of the predicted TSS; For *qrr*4, two possible LuxO-binding sites were identified, located at -103 bp and -89 bp upstream of the predicted TSS, respectively. Nucleotides mismatch the consensus sequences of TTGCAW_3_TGCAA (W refers to A/T) were marked in red at corresponding sites (Figure 2A).

To investigate the promoter activity of Qrr1–4, the recombinant pQrr1-, pQrr2-, pQrr3- and pQrr4-*lux* plasmids were constructed by cloning the promoter region of *qrr*1, *qrr*2, *qrr*3, *qrr*4 into plasmid pBBR*lux*, respectively. The relative luminescence activity RLU (LUX/OD_600_) of recombinant pQrr1-, pQrr2-, pQrr3- and pQrr4-*lux* plasmids was measured in WT, LuxO deletion strain Δ*luxO* [19] and LuxO point mutation strain *luxO*D47E, with higher RLU reflecting stronger promoter activity. The D47E mutation alters the site of phosphorylation and “locks” the LuxO protein into a phosphorylated state, mimicking LuxO∼P [11]. As shown in Figure 2B, in WT, the RLUs of pQrr4-*lux* were significantly stronger than the RLUs of pQrr1-, pQrr2-, and pQrr3-*lux*, indicating that Qrr4 is the most highly expressed Qrr sRNA. Compared to WT, in Δ*luxO*, the RLUs of pQrr1-, pQrr2-, and pQrr3-*lux* decreased significantly and were hardly detectable. For pQrr4-*lux*, its RLUs decreased less than fourfold. In *luxO*D47E, the RLUs of pQrr1-, pQrr2-, and pQrr3-*lux* increased remarkably, with the values of RLU rising to above 100,000. For pQrr4-*lux*, its RLUs in *luxO*D47E were similar to its RLUs in WT.

To further demonstrate the role of LuxO on the promoter activity of Qrr sRNAs in *V*. *fluvialis*, site-directed mutagenesis was introduced into each putative LuxO-binding site by mutating the conserved half-site of the palindromic sequence (Figure 2A). Recombinant pQrr1-, pQrr2-, pQrr3-, and pQrr4-*lux* mutant plasmids were constructed and then introduced into WT, Δ*luxO*, and *luxO*D47E, respectively, to measure their luminescence activity (Figure 2C–E). As shown in Figure 2C, in WT, mutations of putative LuxO-binding sites decreased the luminescence activity of mutant plasmids compared to their corresponding wildtype plasmids. For Qrr1, 2, and 4, the effect of simultaneously mutating two putative LuxO-binding sites was similar to that of only mutating one putative LuxO-binding site. The results in *luxO*D47E (Figure 2E) were similar to those in WT. By contrast, the deficiency of luminescence activity caused by potential LuxO-binding site mutation was less significant in Δ*luxO* (Figure 2D). Together, these results showed that LuxO is essential for the promoter activity of Qrr1–4, and mutation of putative LuxO-binding sites caused deficiency in their promoter activity.

### The small RNA chaperone Hfq is involved in VflT6SS2 modulation

**Figure 3.**
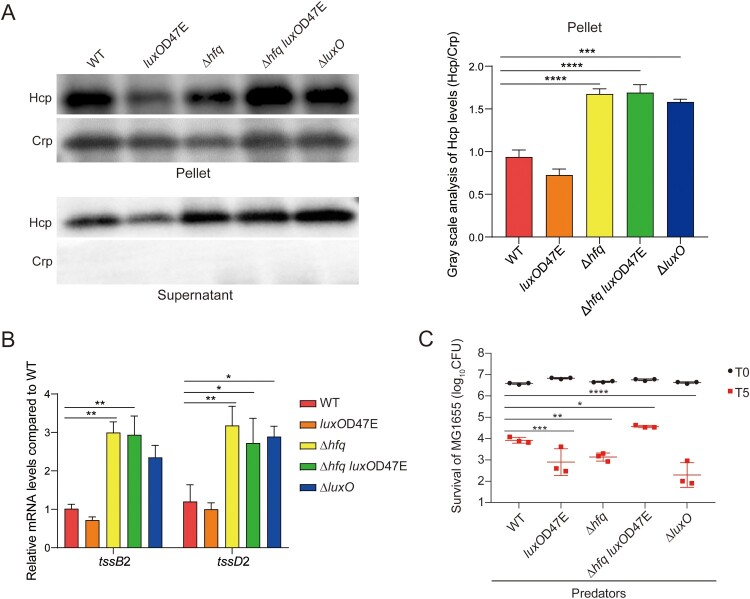
The deletion of RNA chaperone Hfq influences the Hcp expression, secretion and bacterial killing ability of *V*. *fluvialis*. (A) Hcp expression and secretion in WT, *luxO*D47E, Δ*hfq*, Δ*hfq luxO*D47E, and Δ*luxO* strains. Strains were incubated at 30°C in LB medium to OD_600_ of 0.5–0.8. Western blot analysis was performed with protein extract from cell pellets and supernatants. Crp protein was used as internal control. The graph shows the gray scale of Hcp protein relative to Crp protein. The data represent the means ± standard error of mean (SEM) of three independent experiments. One-way ANOVA with Dunnett correction for multiple testing; asterisks represent a significant difference from WT as follows: ***, *P *< 0.001; ****, *P *< 0.0001. (B) The relative mRNA levels of *tssB*2 and *tssD*2 in WT, *luxO*D47E, Δ*hfq*, Δ*hfq luxO*D47E strains. The strains were cultured to OD_600_ of 0.2 and the total RNA was extracted. The *tssB*2 and *tssD*2 mRNA levels were measured by qRT-PCR. The *recA* mRNA levels were used as internal control. The data represent the means ± standard error of mean (SEM) of three independent biological replicates. Two-way ANOVA with Dunnett correction for multiple testing; asterisks represent a significant difference from WT as follows: *, *P *< 0.05; **, *P *< 0.01. (C) Bacterial killing assay was performed between *V*. *fluvialis* (WT or mutant) strain and *E*. *coli* MG1655 strain. The CFU of the prey MG1655 was calculated at the start point (T0) and after 5 h (T5) co-culture with corresponding predator. The data represent the means ± standard error of mean (SEM) of three independent biological replicates. Two-way ANOVA with Dunnett correction for multiple testing; asterisks represent a significant difference from WT as follows: *, *P *< 0.05; **, *P *< 0.01; ***, *P *< 0.001; ****, *P *< 0.0001.

In many bacteria, the small RNA chaperone Hfq is required for efficient base pairing between an sRNA and its target mRNA [28]. Because the *qrr* genes in *V*. *fluvialis* locate in intergenic regions, the deletion of *qrr* genes may influence the expression of their downstream genes. Therefore, we constructed Hfq deletion strains Δ*hfq* and Δ*hfq luxO*D47E to explore the role of Qrr sRNAs on VflT6SS2 modulation. We first detected the levels of Hcp protein, a hallmark of T6SS function, in the cell pellets and supernatants of WT, Δ*luxO*, *luxO*D47E, Δ*hfq* and Δ*hfq luxO*D47E strains. As shown in Figure 3A, compared to WT, the levels of Hcp protein in pellets were increased in Δ*luxO* but decreased in *luxO*D47E, where the Qrr sRNAs are in a transcriptionally repressed and activated state, respectively (Figure 2D and E), indicating the Qrr sRNAs probably participate in downregulation of VflT6SS2. Consistently, the levels of Hcp protein in pellets were also increased in the absence of Hfq, supporting the Hfq-dependent repression effect of Qrr sRNAs on VflT6SS2. To better compare the differences in Hcp expression among the different genetic groups, we presented a gray scale analysis plot of the western blot bands of three independent repetitions (Figure 3A). The intensities of Hcp bands normalized to the internal reference Crp were increased in Δ*luxO*, Δ*hfq*, and Δ*hfq luxO*D47E, but slightly decreased in *luxO*D47E relative to WT. Similar trend was observed in supernatants, compared to WT, the secretion levels of Hcp protein were increased in Δ*luxO*, Δ*hfq*, and Δ*hfq luxO*D47E but decreased in *luxO*D47E. For further confirmation, we measured the mRNA expression levels of *tssB*2 and *tssD*2, two representative genes of VflT6SS2 at LCD (OD_600_ of 0.2) when the Qrr sRNAs are actively expressed. As shown in Figure 3B, compared to WT, the *tssB*2 and *tssD*2 mRNA abundances were increased in Δ*luxO*, Δ*hfq*, and Δ*hfq luxO*D47E, but slightly decreased in *luxO*D47E. The less significant difference between WT and *luxO*D47E was not surprising considering the activated state of Qrr sRNAs in WT at LCD.

We further explored the role of Qrr sRNAs on VflT6SS2 modulation by bacterial killing assay. The bactericidal ability of WT, Δ*luxO*, *luxO*D47E, Δ*hfq* and Δ*hfq luxO*D47E were examined by measuring the survival of *E*. *coli* MG1655 strain (prey) after co-culture with corresponding *V*. *fluvialis* strain (predator). As shown in Figure 3C, after 5 h incubation, compared to WT, the number of survived MG1655 was slightly increased in *luxO*D47E, but noticeably decreased in Δ*luxO*, Δ*hfq*, and Δ*hfq luxO*D47E. Together, these results demonstrated that the sRNAs chaperone Hfq is involved in the function of VflT6SS2, suggesting the importance of Qrr sRNAs towards VflT6SS2 modulation.

### Qrr4 represses QS regulator HapR through direct base pairing to the 5′ UTR of mRNA

**Figure 4.**
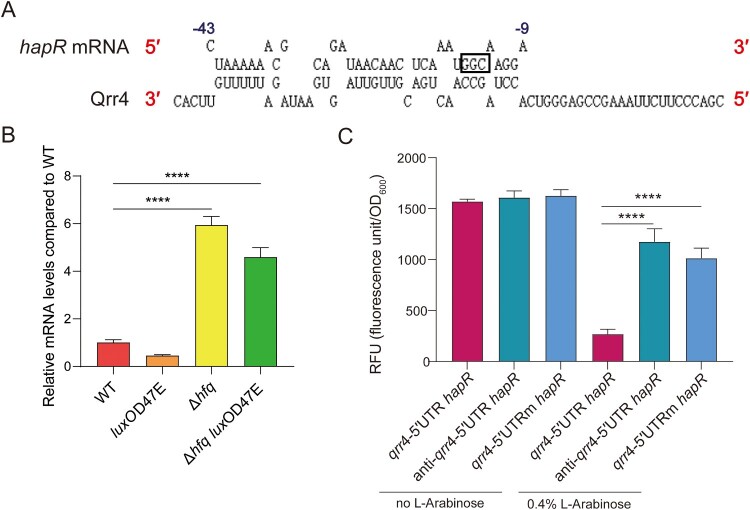
Qrr4 targets the QS regulator HapR. (A) Prediction of base-pairing formed by Qrr4 and the 5′ UTR of *hapR* mRNA. The 3′ and 5′ ends are labelled by red. For *hapR* mRNA, the nucleotide positions of the 5′ UTR upstream of the start codon are shown. The three-nucleotide mutation in the 5′ UTR of *hapR* mRNA is labelled by box. (B) The relative mRNA levels of HapR in WT, *luxO*D47E, Δ*hfq*, Δ*hfq luxO*D47E strains. The strains were cultured to OD_600_ of 1.5 and the total RNA was extracted. The *hapR* mRNA levels were measured by qRT-PCR. The *recA* mRNA levels were used as internal control. The data represent the means ± standard error of mean (SEM) of three independent biological replicates. One-way ANOVA with Tukey correction for multiple testing; asterisks represent a significant difference from WT as follows: ****, *P *< 0.0001. (C) The fluorescence activity of recombinant pET-28a-EGFP-c translation fusion plasmids containing wild-type or mutant 5′ UTR of *hapR* co-cultured with recombinant pBAD33 plasmids containing *qrr*4 or anti-*qrr*4 in *E*. *coli* BL21. The BL21 strains harbouring corresponding recombinant pET-28a-EGFP-c and pBAD33 plasmids were cultured to OD_600_ of 0.4∼0.6. 0.01 mM IPTG was added for induction with or without 0.4% L-Arabinose. 200 μL bacterial suspension was transferred to an opaque-wall 96-well microtiter plate to measure the fluorescence units. The relative fluorescence units RFU (fluorescence unit/OD_600_) were then calculated. The data represent the means ± standard error of mean (SEM) of three independent biological replicates. One-way ANOVA with Tukey correction for multiple testing; asterisks represent a significant difference from WT as follows: ****, *P *< 0.0001.

Above we have shown that LuxO is essential for the promoter activity of Qrr sRNAs (Figure 2B–E). In QS system, Qrr sRNAs have been shown to function between LuxO and the downstream QS regulator, such as LuxR in *V*. *harveyi* or HapR in *V*. *cholerae* [11]. Many sRNAs act by base pairing to complementary regions in the 5′ UTR of the target mRNA [11]. Therefore, we investigated whether Qrr sRNAs can directly target HapR in *V*. *fluvilais*. We first aligned the 5′ UTR of *hapR* mRNA and the sequence of Qrr4, which is found to be the most highly expressed Qrr sRNA (Figure 2B–E), to identify potential base-pairing. A putative region of 6 + 1 + 2 + 7 + 3 + 4 + 3 base-pair interaction interrupted with internal bulged-out nucleotides was suggested between the Qrr4 and the 5′ UTR of *hapR* mRNA (Figure 4A). Results showed that the regions being potentially involved in the complementary base pairing are highly conserved among all four Qrr sRNAs in *V*. *fluvilais* and implicate the SL2 and SL3 of the predicted secondary structure of Qrr4 (Figure 1).

We next investigated the *hapR* mRNA levels in WT, Δ*hfq*, *luxO*D47E, and Δ*hfq luxO*D47E. As shown in Figure 4B, compared to WT and *luxO*D47E, the deletion of Hfq significantly increased the *hapR* mRNA levels in Δ*hfq* and Δ*hfq luxO*D47E. Furthermore, we used GFP reporter assay to examine the role Qrr4 plays in *hapR* regulation in a heterologous system by generating 5′ UTR HapR-GFP translational fusion (−80 to +18 with +1 as the translational start site). We constructed WT pET-5′ UTR *hapR*-EGFP and mutant pET-5′ UTRm *hapR*-EGFP (containing three-nucleotide mutation in the Qrr4-*hapR* 5′ UTR binding site, as shown in Figure 4A) translation fusion plasmids based on the pET-28a-EGFP-c vector which is IPTG induced. *qrr*4 and anti-*qrr*4 (the antisense sequence of *qrr*4) overexpression plasmids were also constructed in the background of L-arabinose-induced expression vector pBAD33. Then the recombinant GFP translation fusion plasmid and the *qrr*4 or anti-*qrr*4 overexpression plasmids were introduced into *E*. *coli* BL21. Under the induction of IPTG together with or without L-Arabinose, the relative fluorescence units (RFUs), which indicate the translational levels of *hapR*, were measured. As shown in Figure 4C, without the induction of L-Arabinose, the RFUs of *qrr*4-5′ UTR *hapR*, anti-*qrr*4-5′ UTR *hapR* and *qrr*4-5′ UTRm *hapR* were similarly high. However, when L-Arabinose was added, the RFUs of *qrr*4-5′ UTR *hapR* dropped significantly (almost 6.5-fold), indicating *qrr*4 is sufficient to suppress translation of transcripts harbouring the 5′ UTR of *hapR*. By contrast, mutation of GGC in the 5′ UTR of *hapR* to its complementary bases CCG greatly compromised the repression effect of *qrr*4, with the light output similar to the control condition with the reverse complement of *qrr*4, anti-*qrr*4, which theoretically possesses no significant complementarity to the *hapR* 5′ UTR. We also noticed the mild RFU signal decreases for anti-*qrr*4-5′ UTR *hapR* and *qrr*4-5′ UTRm *hapR* when compared to *qrr*4-5′ UTR *hapR*. We speculated the possible reasons for these reductions. For *qrr*4-5′ UTRm *hapR*, the GGC mutation in the 5′ UTR of *hapR* may mainly affect the binding affinity of Qrr4 for *hapR* instead of completely abolishing its binding. Therefore, the mild repression was observed when *qrr*4 was induced to express in large quantities. While for anti-*qrr*4-5′ UTR *hapR*, limited complementarities may potentially exist and remain to be confirmed in the future. Together, these results indicated that Qrr4 can directly interact with the 5′ UTR of *hapR* mRNA and repress the expression of HapR.

### Qrr4 also targets the 5′ UTR of the mRNA of tssB2 (vipA) and tssD2_a (hcp) of VflT6SS2 through direct base pairing

**Figure 5.**
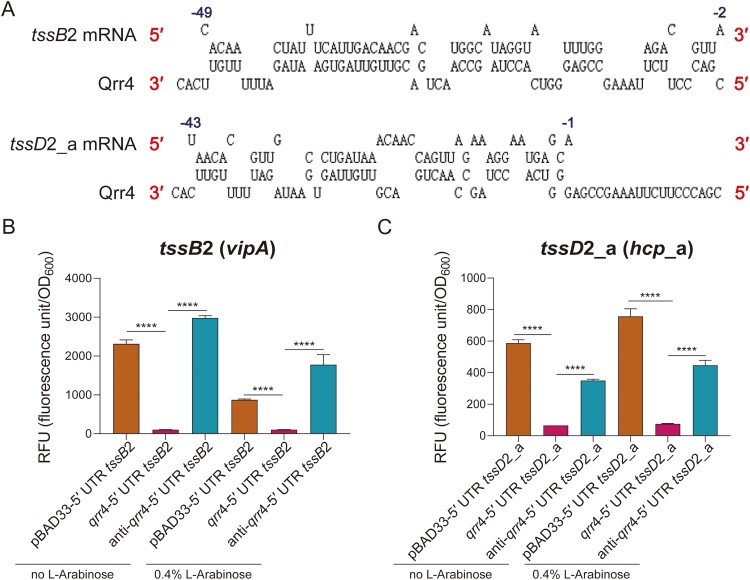
Qrr4 targets the *tssB*2 (*vipA*) and *tssD*2_a (*hcp*_a) genes of VflT6SS2. (A) Prediction of base-pairing formed by Qrr4 and the 5′ UTR of *tssB*2 or *tssD*2_a mRNA. The 3′ and 5′ ends are labelled by red. For *tssB*2 or *tssD*2_a mRNA, the nucleotide positions of the 5′ UTR upstream of the start codon are shown. (B–C) The fluorescence activity of recombinant pET-28a-EGFP-c translation fusion plasmids containing wild-type 5′ UTR of *tssB*2 (B) or *tssD*2_a (C) co-cultured with recombinant pBAD33 plasmids containing *qrr*4 or anti-*qrr*4 in *E*. *coli* BL21. The fluorescence activity recombinant pET-28a-EGFP-c translation fusion plasmids co-cultured with empty pBAD33 plasmid was used as negative control. The experimental procedure and the calculation of RFU are the same as Figure 4C. The data represent the means ± standard error of mean (SEM) of three independent biological replicates. One-way ANOVA with Tukey correction for multiple testing; asterisks represent a significant difference from WT as follows: ****, *P *< 0.0001.

In *V*. *cholerae*, Qrr sRNAs can not only repress HapR, but also repress the major cluster of T6SS through base pairing [18]. To investigate whether Qrr4 can directly regulate the VflT6SS2 in *V*. *fluvialis*, we firstly aligned the 5′ UTR mRNA sequences of *tssB*2 (*vipA*) and *tssD*2_a (*hcp*_a) and the sequence of Qrr4 to identify potential base-pairing (Figure 5A). *tssB*2 (*vipA*) is the first gene of the major cluster of VflT6SS2 while *tssD*2_a (*hcp*_a) is the highly transcriptional one among the two units of the first auxiliary cluster of VflT6SS2 [29]. Discontinuous interactions of 4 + 4 + 12 + 1 + 4 + 5 + 5 + 3 + 3 and 4 + 3 + 1 + 7 + 5 + 1 + 3 + 3 base pairing with Qrr4 were revealed for *tssB*2 (*vipA*) and *tssD*2_a (*hcp*_a) respectively (Figure 5A). Besides, results showed that the regions being potentially involved in the complementary base pairing with the 5′ UTR mRNA sequences of *tssB*2 (*vipA*) and *tssD*2_a (*hcp*_a) mostly locate in the highly conserved regions among all four Qrr sRNAs in *V*. *fluvilais* (Figure 1). Unlike the base-pairing with the 5′ UTR of *tssB*2 (*vipA*) mRNA, which involves the SL2 and SL3 of the predicted secondary structure of Qrr4, the base-pairing with the 5′ UTR of *tssD*2_a (*hcp*_a) mRNA implicates the SL1, SL2, and SL3 (Figure 1).

We further constructed WT pET-5′ UTR *tssB*2-EGFP and pET-5 ′UTR *tssD*2_a-EGFP translation fusion plasmids in the background of pET-28a-EGFP-c vector. Then the recombinant GFP translation fusion plasmid and the *qrr*4 or anti-*qrr*4 overexpression plasmids were introduced into *E*. *coli* BL21, as above mentioned. Under the induction of IPTG together with or without L-Arabinose, the RFUs were measured. As shown in Figure 5B and C, compared to pBAD33-5′ UTR *tssB*2 and pBAD33-5′ UTR *tssD*2_a, the RFUs of *qrr*4-5′ UTR *tssB*2 and *qrr*4-5′ UTR *tssD*2_a dropped greatly even without the induction of L-Arabinose, while the introduction of anti-*qrr*4 failed to cause the decrease of RFUs of corresponding GFP translation fusion plasmids. We speculated that the Qrr4-related down-regulation phenotype under uninduced condition might be due to the leaky expression of P_BAD_ promoter which has been reported in the literature [30]. Another contributing reason is that low level of Qrr4 is sufficient for the regulation of *tssB*2 and *tssD*2_a, probably originated from more efficient base-pairing interactions. Interestingly but perhaps surprisingly, for *tssB*2, the addition of L-Arabinose in general resulted a decrease of the levels of RFU in relative to the absence of inducer, which was different from *tssD*2_a. Together, these results indicated that Qrr4 can directly form base-pairing with the 5′ UTR mRNAs of *tssB*2 and *tssD*2_a and specifically repress the expression of *tssB*2 and *tssD*2_a.

## Discussion

In this study, we aimed to characterize the Qrr sRNAs and their roles on VflT6SS2 modulation in *V*. *fluvialis*. We found that there are four Qrr sRNAs (Qrr1–4) in *V*. *fluvialis*, each of them has one or two LuxO-binding site(s) in the promoter region upstream of the predicted TSS, indicating the regulation by LuxO. Also, the four Qrr sRNAs show different magnitude of expression, which can be affected by the absence or phosphorylation of LuxO and the single or combined mutation of LuxO-binding site(s). Qrr4, the most highly expressed Qrr sRNA, can directly down-regulate the expression of *hapR* as well as the VflT6SS2 genes post-transcriptionally. Consistently, deletion of Hfq, the sRNA chaperone, leads to the increase of *hapR*, *tssB*2, and *tssD*2 mRNA levels and enhances the function of VflT6SS2 in terms of its secretion and bactericidal activities. We propose that like other *Vibrio* species, Qrr sRNAs and Hfq in *V*. *fluvialis* can function between LuxO and HapR to regulate VflT6SS2. Notably, Qrr4 can directly repress not only HapR, but also the major as well as auxiliary VflT6SS2 gene clusters, making the repression towards VflT6SS2 more complex and refined.

Being a kind of Hfq-dependent *trans*-encoded sRNAs that control QS, Qrr sRNAs have been found in various *Vibrio* species [31]. The number of Qrr sRNAs varies in different *Vibrio* species but their sequences share similar characteristics [11]. In this study, we found that the number of *qrr* locus in *V*. *fluvialis* is four, the same as that in *V*. *cholerae*. According to previous studies, a pattern exists between the number of putative *qrr* family members and the evolutionary history of Vibrionaceae [32]. Research on the evolution and phylogenetic relationships of *Vibrio* demonstrated that *V*. *cholerae* and *V*. *fluvialis* are in the Cholerae clade [33], which may explain the identical number of their Qrr sRNAs. In this study, we found that one Qrr sRNA (Qrr4) is higher expressed than the others, consistent with the findings in *V*. *harveyi* and *V*. *cholerae* [11]. Previous studies have found that in *Vibrionaceae*, for species with multiple *qrr* loci, one *qrr* copy locates upstream of the *luxO* locus on the large chromosome and the remaining copies spread across the small chromosome [32]. Our study in *V. fluvialis* confirmed this as *qrr*1 locates next to the *luxO* locus on the large chromosome and *qrr*2–3 are on the small chromosome.

In *V*. *cholerae*, *V*. *harveyi*, and *V*. *vulnificus*, the consensus LuxO-binding sequence (5′-TTGCAW_3_TGCAA-3′) is present in the promoter regions of all *qrr* genes [11,27,34], consistent with our findings about *V*. *fluvialis*. The number, location and conservation of the consensus LuxO-binding sequence vary in different *qrr* genes, which is considered to influence the activation of *qrr* transcription by LuxO. According to the patterns of the LuxO-binding sequences of the highest expressed *qrr* in *V*. *harveyi* (*qrr*4), *V*. *vulnificus* (*qrr*2), and *V*. *fluvialis* (*qrr*4), we found some characteristics in common: (i) more than one LuxO-binding sites are needed and at least one should be perfectly matched to the consensus sequence; (ii) the LuxO-binding sites should be close to each other. Our results about the mutation in the LuxO-binding sites of *qrr* genes showed that for *qrr* genes with two different LuxO-binding sites, the two sites do not exert additive effect since the mutagenesis of either site can abolish the regulatory effect of LuxO and cause repression in the *qrr* promoter activity. As LuxO likely activates transcription through a DNA looping mechanism [35], we propose that some specific DNA secondary structures formed by both LuxO-binding sites are needed for the maximum transcriptional activation of *qrr* genes by LuxO.

**Figure 6.**
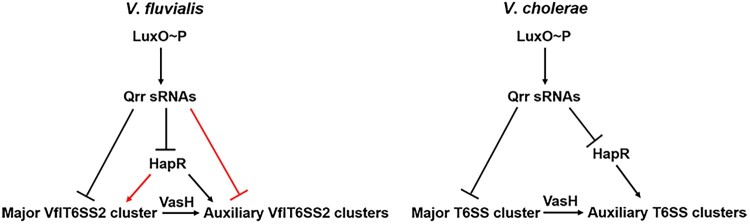
Model for Qrr sRNA control of VflT6SS2 in *V*. *fluvialis*. Phosphorylated LuxO (LuxO∼P) activates the production of Qrr sRNAs, which is the same in both *V*. *fluvialis* and *V*. *cholerae*. However, different from *V*. *cholerae* (on the right), in *V*. *fluvialis* (on the left), Qrr sRNAs not only directly repress HapR and the major VflT6SS2 (T6SS for *V*. *cholerae*) cluster, but also directly repress the auxiliary VflT6SS2 clusters (labelled by red). In *V*. *fluvialis*, HapR then activates the expression of the major (labelled by red) and auxiliary VflT6SS2 clusters, while in *V*. *cholerae*, HapR only activates the auxiliary T6SS clusters. The function of VasH is the same in *V*. *fluvialis* and *V*. *cholerae*, as it only activates the auxiliary VflT6SS2 (T6SS) clusters.

In *V*. *cholerae*, the QS response regulator LuxO represses T6SS at LCD and the QS master regulator HapR activates T6SS at HCD [18]. Qrr sRNAs are needed to function between LuxO and HapR in the QS cascade and exert regulatory effect through two mechanisms. On the one hand, Qrr sRNAs directly repress expression of the major T6SS cluster through base pairing. On the other hand, Qrr sRNAs repress HapR, the activator of the two auxiliary T6SS clusters. The regulatory arrangement of Qrr sRNAs in *V*. *cholerae* ensures that the major cluster encoding many components of the secretory machine is expressed prior to the two auxiliary clusters encoding the secreted effectors, guaranteeing the efficient secretion of effectors after synthesis. In *V*. *fluvialis*, the CqsA/LuxS-HapR QS regulator LuxO represses while HapR activates VflT6SS2, which is functionally expressed [21]. In this study, we found that like the QS regulation in *V*. *cholerae*, Qrr sRNAs also function between LuxO and HapR in *V*. *fluvialis*. However, in *V*. *fluvialis*, the regulation of Qrr sRNAs toward T6SS is more complicated than that in *V*. *cholerae* (Figure 6). In *V*. *fluvialis*, Qrr sRNAs can repress not only HapR, but also the major and auxiliary VflT6SS2 clusters. Unlike *V*. *cholerae*, HapR in *V*. *fluvialis* activates not only the three auxiliary VflT6SS2 clusters, but also the major VflT6SS2 cluster [21]. We speculated that the Qrr sRNAs and HapR mediated multilayer coregulation of the major and auxiliary clusters favours the fine tuning of VflT6SS2 according to the growth status of cells: keeping the strong repression at LCD, gradually quick activation along with cell growth and achieving maximum expression at HCD. In VflT6SS2, the auxiliary clusters are responsible for the production of Hcp protein and effectors while the major cluster for the structural proteins together with a transcriptional activator VasH, which then assembles into secretion machine to secret Hcp and effector proteins [20]. The Qrr sRNAs and HapR mediated regulation on VflT6SS2 major cluster also affect the VasH level which is strictly required for Hcp expression [36], thus executing another layer of regulation on the VflT6SS2. For *Vibrio* species possessing multiple Qrr sRNAs, not every Qrr sRNA is necessary or equally important. In this study, Qrr4 was used as a representative. The role of other Qrr sRNAs on VflT6SS2 and how the four Qrr sRNAs function need further exploration. Among the five Qrr sRNAs in *V*. *harveyi*, only four are required for QS behaviours. These four Qrr sRNAs function additively and have hierarchical roles as follows: Qrr4 > Qrr2 > Qrr3 > Qrr1 [27]. However, in *V*. *cholerae*, the four Qrr sRNAs are completely redundant, since only the simultaneous deletion of all four *qrr* genes eliminates the effect on QS-controlled behaviours and any one of the four Qrr sRNAs is sufficient to maintain QS dependent gene expression [11,18].

In conclusion, our study characterizes the Qrr sRNAs in *V*. *fluvialis* and their roles toward VflT6SS2 modulation. Qrr sRNAs are regulated by LuxO and repress the downstream QS master regulator HapR. Qrr sRNAs also directly repress both the major and the auxiliary VlfT6SS2 clusters. These findings provide important insights toward the QS and T6SS regulation in *V*. *fluvialis* and enhance understandings of this emerging foodborne pathogen.

## Supplementary Material

Supplementary material.docx
