# Supplementary material for: Characterization of quorum regulatory small RNAs in an emerging pathogen Vibrio fluvialis and their roles toward type VI secretion system VflT6SS2 modulation
Source: Emerg Microbes Infect. 2024 Aug 28;13(1):2396872. doi: 10.1080/22221751.2024.2396872 (PMC11443567; doi:10.1080/22221751.2024.2396872)
Supplement: Supplementary material.docx [file TEMI_A_2396872_SM6183.docx]

**Table S1. Bacterial strains used in this study.**

| **Strains** | **Relevant Characteristics** | **Reference** |
| --- | --- | --- |
| ***V. fluvialis*** |  |  |
| 85003 (Wild type) | Clinical isolate, Sm^R^ | [1] |
| Δ*hfq* | 85003 with *hfq* in-frame deletion, Sm^R^ | This study |
| ∆*luxO* | 85003 with *luxO* in-frame deletion, Sm^R^ | [2] |
| *luxO*D47E | 85003 with *luxO* D47E mutation, Sm^R^ | This study |
| Δ*hfq luxO*D47E | 85003 with *hfq* in-frame deletion and *luxO* D47E mutation, Sm^R^ | This study |
| pQrr1-*lux*/85003 | strain 85003 carrying the plasmid pQrr1-*lux*, Sm^R^ | This study |
| pQrr2-*lux*/85003 | strain 85003 carrying the plasmid pQrr2-*lux*, Sm^R^ | This study |
| pQrr3-*lux*/85003 | strain 85003 carrying the plasmid pQrr3-*lux*, Sm^R^ | This study |
| pQrr4-*lux*/85003 | strain 85003 carrying the plasmid pQrr4-*lux*, Sm^R^ | This study |
| pQrr1-*lux*/Δ*luxO* | strain ∆*luxO* carrying the plasmid pQrr1-*lux*, Sm^R^ | This study |
| pQrr2-*lux*/Δ*luxO* | strain ∆*luxO* carrying the plasmid pQrr2-*lux*, Sm^R^ | This study |
| pQrr3-*lux*/Δ*luxO* | strain ∆*luxO* carrying the plasmid pQrr3-*lux*, Sm^R^ | This study |
| pQrr4-*lux*/Δ*luxO* | strain ∆*luxO* carrying the plasmid pQrr4-*lux*, Sm^R^ | This study |
| pQrr1-*lux*/*luxO*D47E | strain *luxO* D47E carrying the plasmid pQrr1-*lux*, Sm^R^ | This study |
| pQrr2-*lux*/*luxO*D47E | strain *luxO* D47E carrying the plasmid pQrr2-*lux*, Sm^R^ | This study |
| pQrr3-*lux*/*luxO*D47E | strain *luxO* D47E carrying the plasmid pQrr3-*lux*, Sm^R^ | This study |
| pQrr4-*lux*/*luxO*D47E | strain *luxO* D47E carrying the plasmid pQrr4-*lux*, Sm^R^ | This study |
| ***E. coli*** |  |  |
| SM10*λpir* | *thr thi tonA leu supE lacY recA*: :RP4-2Tc : :Mu (*λpir*R6K), Km^R^ | Laboratory stock |
| DH5α*λpir* | F^-^D(*lacZYA*-*argF*) U169*recA* *endA*1 *supE*44 *relA*1 *λ*::*pir* | Laboratory stock |
| BL21 (DE3) | F- ompT hsdS (rB- mB-) gal dcm (DE3) | Laboratory stock |
| MG1655 | K^-^12 F^–^ λ^–^ *ilvG*^–^ *rfb*-50 *rph*-1, Rfp^R^ | Laboratory stock |
| *qrr*4-5′ UTR *hapR*/BL21 | strain BL21 carrying plasmids *qrr*4-pBAD33 and pET-5′ UTR *hapR*-EGFP, Cm^R^Km^R^ | This study |
| anti-*qrr*4-5′ UTR *hapR*/BL21 | strain BL21 carrying plasmids anti-*qrr*4-pBAD33 and pET-5′ UTR *hapR*-EGFP, Cm^R^Km^R^ | This study |
| *qrr*4-5′ UTRm *hapR*/BL21 | strain BL21 carrying plasmids *qrr*4-pBAD33 and pET-5′ UTRm *hapR*-EGFP, Cm^R^Km^R^ | This study |
| pBAD33-5′ UTR *tssB*2/BL21 | strain BL21 carrying plasmids pBAD33 and pET-5′ UTR *tssB*2-EGFP, Cm^R^Km^R^ | This study |
| *qrr*4-5′ UTR *tssB*2/BL21 | strain BL21 carrying plasmids *qrr*4-pBAD33 and pET-5′ UTR *tssB*2-EGFP, Cm^R^Km^R^ | This study |
| anti-*qrr*4-5′ UTR *tssB*2/BL21 | strain BL21 carrying plasmids anti-*qrr*4-pBAD33 and pET-5′ UTR *tssB*2-EGFP, Cm^R^Km^R^ | This study |
| pBAD33-5′ UTR *tssD*2_a/BL21 | strain BL21 carrying plasmids pBAD33 and pET-5′ UTR *tssD*2_a-EGFP, Cm^R^Km^R^ | This study |
| *qrr*4-5′ UTR *tssD*2_a/BL21 | strain BL21 carrying plasmids *qrr*4-pBAD33 and pET-5′ UTR *tssD*2_a-EGFP, Cm^R^Km^R^ | This study |
| anti-*qrr*4-5′ UTR *tssD*2_a/BL21 | strain BL21 carrying plasmids anti-*qrr*4-pBAD33 and pET-5′ UTR *tssD*2_a-EGFP, Cm^R^Km^R^ | This study |

**Table S2. Plasmids used in this study.**

| **Plasmids** | **Relevant Characteristics** | **Reference** |
| --- | --- | --- |
| pBBR*lux* | bioluminescence based reporter plasmid containing a promoterless *luxCDABE* operon, Cm^R^ | Laboratory stock |
| pWM91 | Suicide vector containing R6K *ori*, *sacB* and *lacZ*α, Amp^R^ | Laboratory stock |
| pWM-Δ*hfq* | pWM91 containing the flanking region sequence of *hfq* | This study |
| pWM-*luxO*D47E | pWM91 containing the sequence of *luxO* with D47E point mutation | This study |
| pQrr1-*lux* | pBBR*lux* containing the promoter region of *qrr*1 | This study |
| pQrr2-*lux* | pBBR*lux* containing the promoter region of *qrr*2 | This study |
| pQrr3-*lux* | pBBR*lux* containing the promoter region of *qrr*3 | This study |
| pQrr4-*lux* | pBBR*lux* containing the promoter region of *qrr*4 | This study |
| pQrr1mut1-*lux* | pBBR*lux* containing mutant promoter region of *qrr*1 (mutation in the putative LuxO-binding site 1) | This study |
| pQrr1mut2-*lux* | pBBR*lux* containing mutant promoter region of *qrr*1 (mutation in the putative LuxO-binding site 2) | This study |
| pQrr1mut1/2-*lux* | pBBR*lux* containing mutant promoter region of *qrr*1 (mutation in the putative LuxO-binding site 1 and 2) | This study |
| pQrr2mut1-*lux* | pBBR*lux* containing mutant promoter region of *qrr*2 (mutation in the putative LuxO-binding site 1) | This study |
| pQrr2mut2-*lux* | pBBR*lux* containing mutant promoter region of *qrr*2 (mutation in the putative LuxO-binding site 2) | This study |
| pQrr2mut1/2-*lux* | pBBR*lux* containing mutant promoter region of *qrr*2 (mutation in the putative LuxO-binding site 1 and 2) | This study |
| pQrr3mut-*lux* | pBBR*lux* containing mutant promoter region of *qrr*3 (mutation in the putative LuxO-binding site) | This study |
| pQrr4mut1-*lux* | pBBR*lux* containing mutant promoter region of *qrr*4(mutation in the putative LuxO-binding site 1) | This study |
| pQrr4mut2-*lux* | pBBR*lux* containing mutant promoter region of *qrr*4 (mutation in the putative LuxO-binding site 2) | This study |
| pQrr4mut1/2-*lux* | pBBR*lux* containing mutant promoter region of *qrr*4 (mutation in the putative LuxO-binding site 1 and 2) | This study |
| pBAD33 | Cloning vector with pBAD promoter, p15A *ori, araC*, Cm^R^ | Laboratory stock |
| pET-28a-EGFP-c | GFP reporter plasmid, Km^R^ | Laboratory stock |
| *qrr*4-pBAD33 | pBAD33 containing *qrr*4 coding fragment | This study |
| anti-*qrr*4-pBAD33 | pBAD33 containing the antisense fragment of *qrr*4 | This study |
| pET-5′ UTR *hapR*-EGFP | pET-28a-EGFP containing the 5′ UTR fragment of *hapR* | This study |
| pET-5′ UTRm *hapR*-EGFP | pET-28a-EGFP containing the 5′ UTR mutant fragment of *hapR* | This study |
| pET-5′ UTR *tssB*2-EGFP | pET-28a-EGFP containing the 5′ UTR fragment of *tssB*2 | This study |
| pET-5′ UTR *tssD*2_a-EGFP | pET-28a-EGFP containing the 5′ UTR fragment of *tssD*2_a | This study |

**Table S3. Primers used in this study.**

| **Primer pairs** | **Sequences (5′-3′)** ^a, b^ | **Amplicon size (bp)** | **Purposes** |
| --- | --- | --- | --- |
| *hfq*-F1-*Xho*Ⅰ | CCGCTCGAGTATCTCGGGCATTGGAAG | 650 (amplify the upstream fragment of the ORF of *hfq*) | Construction of pWM-Δ*hfq* plasmid |
| *hfq*-R1 | GATTTCTCTGCCCCCTTAGCCATTTTATT |  |  |
| *hfq*-F2 | TGGCTAAGGGGGCAGAGAAATCAGAAGAT | 675 (amplify the downstream fragment of the ORF of *hfq*) |  |
| *hfq*-R2-*Sma*Ⅰ | TCCCCCGGGAACCGACTAAGGAAACCG |  |  |
| *luxO*D47E-F1-*BamH*Ⅰ | CGGGATCCCACCACAGCAGTTGGAAA | 568 (amplify the upstream fragment containing *luxO*D47E point mutation) | Construction of pWM-*luxO*D47E plasmid |
| *luxO*D47E-R1 | GCAGGCGCAG**CTC**GAGTAGAATGAGATCCGG |  |  |
| *luxO*D47E-F2 | TCTACTC**GAG**CTGCGCCTGCCGGACATGA | 580 (amplify the downstream fragment containing *luxO*D47E point mutation) |  |
| *luxO*D47E-R2-*Sac*Ⅰ | CGAGCTCGATCGAGATCCATTTCGC |  |  |
| pQrr1-F-*Sac*Ⅰ | ACGAGCTCTACCGTGACACGTAAGCG/ | 512 (amplify the promoter region of *qrr*1) | Construction of pQrr1-*lux* plasmid |
| pQrr1-R-*Spe*Ⅰ | GGACTAGTAAATTGAATAAAGCATATTCCG |  |  |
| pQrr2-F-*Sac*Ⅰ | ACGAGCTCCTCTCTATCGTTGGCCTT/ | 517 (amplify the promoter region of *qrr*2) | Construction of pQrr2-*lux* plasmid |
| pQrr2-R-*Spe*Ⅰ | GGACTAGTGAACTATATAATGCAGATAGC |  |  |
| pQrr3-F-*Sac*Ⅰ | ACGAGCTCTGCGATACCAATTCATGTAC/ | 508 (amplify the promoter region of *qrr*3) | Construction of pQrr3-*lux* plasmid |
| pQrr3-R-*Spe*Ⅰ | GGACTAGTGACTAATATAATGCAGTATATG |  |  |
| pQrr4-F-*Sac*Ⅰ | ACGAGCTCAGCGTCAGAATGCTGGTG/ | 517 (amplify the promoter region of *qrr*4) | Construction of pQrr4-*lux* plasmid |
| pQrr4-R-*Spe*Ⅰ | GGACTAGTGAAATGAATTATGCATTAAGCG |  |  |
| *hapR*-qPCR-F | GGTGGAAGTGTCTGGATGG | 117 | qRT-PCR |
| *hapR*-qPCR-R | TGGCTCAGGTTGGTATGC |  |  |
| *recA*-qPCR-F | ACCGAGTCAACGACGATAAC | 105 |  |
| *recA*-qPCR-R | TGATGAACTGCTGGTGTCTC |  | qRT-PCR |

^a^: The underlined nucleotides indicate corresponding restriction endonuclease sites; ^b^: The bold nucleotides indicate mutation for *luxO*D47E.

**Table S4. Synthetic DNA sequences used in this study.**

| **Synthetic DNA sequences** | **Sequences (5′-3′)** | **Length (bp)** | **Purposes** |
| --- | --- | --- | --- |
| *qrr*1-mut1 | GAGCTCTACCGTGACACGTAAGCGGTCTGCTTCACACGGTTTGATGAGGAAATCCTGTGCGCCATGACGCATCGCCTCAACCGCAGTATCAATTGAACCATGTGCGGTCATAAAAATCACAGGTACATCCGGCGAGCGCTGTTTAACAGCGTGTAATACGTCCATACCGGTCATGTCCGGCAGGCGCAGATCGAGTAGAATGAGATCCGGTTCGCGATGCGCGATACTTTCGATCGCTTCACGCCCAGTTCCGACGATGTTGATATCGATCTCTAGCGGAGTCAGGTACGAGCGATATAACGCCGCCACTGACGCCGTATCTTCGACCATCAGCAGATATTTCGCCTTATGATGAATATCATTTAATTGCATAACCTAGCCATTTATCTTCCATGTTTTGCTTAATAGTCGCATTTGGAGTTGCATTTTGCAAATTCAAAGCGATATTTCCGCAAAAATAGTGTCAGAATTCCAAAGAATCACCGAAATACAAACTGGCACGGAATATGCTTTATTCAATTTACTAGT | 528  (underlined nucleotides indicate restriction endonuclease sites of SacⅠ and SpeⅠ, respectively; boxes indicate putative LuxO-binding site; nucleotides in red indicate mutation) | Construction of pQrr1mut1-*lux* plasmid |
| *qrr*1-mut2 | GAGCTCTACCGTGACACGTAAGCGGTCTGCTTCACACGGTTTGATGAGGAAATCCTGTGCGCCATGACGCATCGCCTCAACCGCAGTATCAATTGAACCATGTGCGGTCATAAAAATCACAGGTACATCCGGCGAGCGCTGTTTAACAGCGTGTAATACGTCCATACCGGTCATGTCCGGCAGGCGCAGATCGAGTAGAATGAGATCCGGTTCGCGATGCGCGATACTTTCGATCGCTTCACGCCCAGTTCCGACGATGTTGATATCGATCTCTAGCGGAGTCAGGTACGAGCGATATAACGCCGCCACTGACGCCGTATCTTCGACCATCAGCAGATATTTCGCCTTATGATGAATATCATTTAATTGCATAACCTAGCCATTTATCTTTTGCATTTTGCTTAATAGTCGCATTTGGAGCCATGTTTTGCAAATTCAAAGCGATATTTCCGCAAAAATAGTGTCAGAATTCCAAAGAATCACCGAAATACAAACTGGCACGGAATATGCTTTATTCAATTTACTAGT | 528  (underlined nucleotides indicate restriction endonuclease sites of SacⅠ and SpeⅠ, respectively; boxes indicate putative LuxO-binding site; nucleotides in red indicate mutation) | Construction of pQrr1mut2-*lux* plasmid |
| *qrr*1-mut1/2 | GAGCTCTACCGTGACACGTAAGCGGTCTGCTTCACACGGTTTGATGAGGAAATCCTGTGCGCCATGACGCATCGCCTCAACCGCAGTATCAATTGAACCATGTGCGGTCATAAAAATCACAGGTACATCCGGCGAGCGCTGTTTAACAGCGTGTAATACGTCCATACCGGTCATGTCCGGCAGGCGCAGATCGAGTAGAATGAGATCCGGTTCGCGATGCGCGATACTTTCGATCGCTTCACGCCCAGTTCCGACGATGTTGATATCGATCTCTAGCGGAGTCAGGTACGAGCGATATAACGCCGCCACTGACGCCGTATCTTCGACCATCAGCAGATATTTCGCCTTATGATGAATATCATTTAATTGCATAACCTAGCCATTTATCTTCCATGTTTTGCTTAATAGTCGCATTTGGAGCCATGTTTTGCAAATTCAAAGCGATATTTCCGCAAAAATAGTGTCAGAATTCCAAAGAATCACCGAAATACAAACTGGCACGGAATATGCTTTATTCAATTTACTAGT | 528  (underlined nucleotides indicate restriction endonuclease sites of SacⅠ and SpeⅠ, respectively; boxes indicate putative LuxO-binding site; nucleotides in red indicate mutation) | Construction of pQrr1mut1/2-*lux* plasmid |
| *qrr*2-mut1 | GAGCTCCTCTCTATCGTTGGCCTTATCTGGTCGCTGCCTGCGGTACCGTTAATGGAATGGCGCGTTGACTGTATCTGGCTGGTTGCGGTGCCGGTTCTTGGTTTCTACTACCGCTTATCCATGACCGTTTTTCTGATGATGCTCGGCTTTACGCTGGCCTGTATCGGGTTGGCCTGGAGTGTGGAACTGATGGCATTGCCTTTATTGCCGCTGTCACTGGCTCTGTTCGTACTGCTGTGGATAGCGCAGTTTGTTGGCCATAAAATTGAAGGCAAGAAACCTTCCTTTCTGAGTGATCTGCAATTCTTATTAATTGGGCCAATCTGGGTGTTCTATAAGCATTAACATGCTGGCCAATTAATCCCATATAACATCCATGATCTGCATATATTCGCAAATTGCAATTCAATTATGATATCTGTTGTAATGAAACTGATGAAAATTTGTCCAGAAAGCGTGTGTTTCAGTAAAAATAAAAGTTGGCACGCTATCTGCATTATATAGTTCACTAGT | 513  (underlined nucleotides indicate restriction endonuclease sites of SacⅠ and SpeⅠ, respectively; boxes indicate putative LuxO-binding site; nucleotides in red indicate mutation) | Construction of pQrr2mut1-*lux* plasmid |
| *qrr*2-mut2 | GAGCTCCTCTCTATCGTTGGCCTTATCTGGTCGCTGCCTGCGGTACCGTTAATGGAATGGCGCGTTGACTGTATCTGGCTGGTTGCGGTGCCGGTTCTTGGTTTCTACTACCGCTTATCCATGACCGTTTTTCTGATGATGCTCGGCTTTACGCTGGCCTGTATCGGGTTGGCCTGGAGTGTGGAACTGATGGCATTGCCTTTATTGCCGCTGTCACTGGCTCTGTTCGTACTGCTGTGGATAGCGCAGTTTGTTGGCCATAAAATTGAAGGCAAGAAACCTTCCTTTCTGAGTGATCTGCAATTCTTATTAATTGGGCCAATCTGGGTGTTCTATAAGCATTAACATGCTGGCCAATTAATCCCATATAACATTTGCAATCTGCATATATTCGCAAATCATGGTTCAATTATGATATCTGTTGTAATGAAACTGATGAAAATTTGTCCAGAAAGCGTGTGTTTCAGTAAAAATAAAAGTTGGCACGCTATCTGCATTATATAGTTCACTAGT | 513  (underlined nucleotides indicate restriction endonuclease sites of SacⅠ and SpeⅠ, respectively; boxes indicate putative LuxO-binding site; nucleotides in red indicate mutation) | Construction of pQrr2mut2-*lux* plasmid |
| *qrr*2-mut1/2 | GAGCTCCTCTCTATCGTTGGCCTTATCTGGTCGCTGCCTGCGGTACCGTTAATGGAATGGCGCGTTGACTGTATCTGGCTGGTTGCGGTGCCGGTTCTTGGTTTCTACTACCGCTTATCCATGACCGTTTTTCTGATGATGCTCGGCTTTACGCTGGCCTGTATCGGGTTGGCCTGGAGTGTGGAACTGATGGCATTGCCTTTATTGCCGCTGTCACTGGCTCTGTTCGTACTGCTGTGGATAGCGCAGTTTGTTGGCCATAAAATTGAAGGCAAGAAACCTTCCTTTCTGAGTGATCTGCAATTCTTATTAATTGGGCCAATCTGGGTGTTCTATAAGCATTAACATGCTGGCCAATTAATCCCATATAACATCCATGATCTGCATATATTCGCAAATCATGGTTCAATTATGATATCTGTTGTAATGAAACTGATGAAAATTTGTCCAGAAAGCGTGTGTTTCAGTAAAAATAAAAGTTGGCACGCTATCTGCATTATATAGTTCACTAGT | 513  (underlined nucleotides indicate restriction endonuclease sites of SacⅠ and SpeⅠ, respectively; boxes indicate putative LuxO-binding site; nucleotides in red indicate mutation) | Construction of pQrr2mut1/2-*lux* plasmid |
| *qrr*3-mut | GAGCTCTGCGATACCAATTCATGTACGCGTTTGCCATCCGTTCGGCTTGGAATTCGGAACGACCACTCTTCCTGAGCGTTGTGCATAATTTCTGGGTATGCAACCCATGGTGCTGCTGTAATCATTGTCGGCCACATATCAATTCAATTTGAACTCTAAACAAATTCCTTTTATAAACTAACAGCTCAAATGAATATTTCAACCCCTAAACTCGACAAAATATTACAGAAAACTCATAAATTTATTTTTTATCAATAAGTTAAGATAGTAAACAACAGTGCACCAAAACAGACAATATATTTAGATGTGTAATTAAAATGACAATTTACTTACAATGACATGTCAGACTGGATAAATAACAAGGCTGTCACTAGCAATTTGAATGAATTTCGCAATTTGCATTCGCAGAACATGGCCGTTACGGCTGAAATAGTGCCAAAAGCAGAGAAAAACGCGCAAGATATAGTCAAAACAGTGCTCTAAAACTTGGCACATATACTGCATTATATTAGTCACTAGT | 520  (underlined nucleotides indicate restriction endonuclease sites of SacⅠ and SpeⅠ, respectively; boxes indicate putative LuxO-binding site; nucleotides in red indicate mutation) | Construction of pQrr3mut-*lux* plasmid |
| *qrr*4-mut1 | GAGCTCAGCGTCAGAATGCTGGTGAAGATGCCGCCGAACAAAGCGATAAGAAACCAGCCGTTAATATGACGAAACGCTGCGGCAAACCCTTCTCGTTTCCACAAACGTAATAAGCTAGGGTTTACCCGACGGATACTTTCGAGCAGGGTATCGTAGACGCCGGTGATAAAAGCAATGGTACCGCCAGATACACCAGGGACGACATCCGCCGCGCCCATCGCCATGCCTTTCAAAAACGTACTAAAGTAATTCATTGAGAACCTTATTCGAACCAACTGAAGTGTGCAGTATAACCAAAAAATCCAGCCAAATCCGATCGATAAATGTGGATCCGCAAAAGTTTCATTCGCACCTTTATAAGCAATTACATGACTATGCAATATGATCATTTCCATGAATTGCAATTATCAATTTGCAAAAAATAAGTCAGGGTTTTCTCAGGAACTGTGTTATTCAGCCCAAAAAAAGGACTTTTAAAGATGGCACGCTTAATGCATAATTCATTTCACTAGT | 513  (underlined nucleotides indicate restriction endonuclease sites of SacⅠ and SpeⅠ, respectively; boxes indicate putative LuxO-binding site; nucleotides in red indicate mutation) | Construction of pQrr4mut1-*lux* plasmid |
| *qrr*4-mut2 | GAGCTCAGCGTCAGAATGCTGGTGAAGATGCCGCCGAACAAAGCGATAAGAAACCAGCCGTTAATATGACGAAACGCTGCGGCAAACCCTTCTCGTTTCCACAAACGTAATAAGCTAGGGTTTACCCGACGGATACTTTCGAGCAGGGTATCGTAGACGCCGGTGATAAAAGCAATGGTACCGCCAGATACACCAGGGACGACATCCGCCGCGCCCATCGCCATGCCTTTCAAAAACGTACTAAAGTAATTCATTGAGAACCTTATTCGAACCAACTGAAGTGTGCAGTATAACCAAAAAATCCAGCCAAATCCGATCGATAAATGTGGATCCGCAAAAGTTTCATTCGCACCTTTATAAGCAATTACATGACTATGCAATATGATCATTTTTGCAAATTGCAATTATCAATTCATGGAAAATAAGTCAGGGTTTTCTCAGGAACTGTGTTATTCAGCCCAAAAAAAGGACTTTTAAAGATGGCACGCTTAATGCATAATTCATTTCACTAGT | 513  (underlined nucleotides indicate restriction endonuclease sites of SacⅠ and SpeⅠ, respectively; boxes indicate putative LuxO-binding site; nucleotides in red indicate mutation) | Construction of pQrr4mut2-*lux* plasmid |
| *qrr*4-mut1/2 | GAGCTCAGCGTCAGAATGCTGGTGAAGATGCCGCCGAACAAAGCGATAAGAAACCAGCCGTTAATATGACGAAACGCTGCGGCAAACCCTTCTCGTTTCCACAAACGTAATAAGCTAGGGTTTACCCGACGGATACTTTCGAGCAGGGTATCGTAGACGCCGGTGATAAAAGCAATGGTACCGCCAGATACACCAGGGACGACATCCGCCGCGCCCATCGCCATGCCTTTCAAAAACGTACTAAAGTAATTCATTGAGAACCTTATTCGAACCAACTGAAGTGTGCAGTATAACCAAAAAATCCAGCCAAATCCGATCGATAAATGTGGATCCGCAAAAGTTTCATTCGCACCTTTATAAGCAATTACATGACTATGCAATATGATCATTTCCATGAATTGCAATTATCAATTCATGGAAAATAAGTCAGGGTTTTCTCAGGAACTGTGTTATTCAGCCCAAAAAAAGGACTTTTAAAGATGGCACGCTTAATGCATAATTCATTTCACTAGT | 513  (underlined nucleotides indicate restriction endonuclease sites of SacⅠ and SpeⅠ, respectively; boxes indicate putative LuxO-binding site; nucleotides in red indicate mutation) | Construction of pQrr4mut1/2-*lux* plasmid |
| *qrr*4 | ACGCGTAACAAAAGTGTCTATAATCACGGCAGAAAAGTCCACATTGATTATTTGCACGGCGTCACACTTTGCTATGCCATAGCATTTTTATCCATAAGATTAGCGGATCCTACCTGACGCTTTTTATCGCAACTCTCTACTGTTTCTCGAGA**CGACCCTTCTTAAAGCCGAGGGTCACCTAGCCAACTGACGTTGTTAGTGAATAGATTTTTGTTCACACCATACATA**AGCCAATCGCGGTTTTGCGATTGGCTCTTTTTTTTCTGGTTAGGTAAGCTT | 279  (underlined nucleotides indicate restriction endonuclease sites of MluⅠ and HindⅡ, respectively; nucleotides in grey indicate the sequences of pBAD promoter; nucleotides in bold indicate the putative base-pairing region of *qrr*4; nucleotides in box indicate the remaining sequence of *qrr*4) | Construction of *qrr*4-pBAD33 plasmid |
| anti-*qrr*4 | ACGCGTAACAAAAGTGTCTATAATCACGGCAGAAAAGTCCACATTGATTATTTGCACGGCGTCACACTTTGCTATGCCATAGCATTTTTATCCATAAGATTAGCGGATCCTACCTGACGCTTTTTATCGCAACTCTCTACTGTTTCTCGAGA**TATGTATGGTGTGAACAAAAATCTATTCACTAACAACGTCAGTTGGCTAGGTGACCCTCGGCTTTAAGAAGGGTCG**AGCCAATCGCGGTTTTGCGATTGGCTCTTTTTTTTCTGGTTAGGTAAGCTT | 279  (underlined nucleotides indicate restriction endonuclease sites of MluⅠ and HindⅡ, respectively; nucleotides in grey indicate the sequences of pBAD promoter; nucleotides in bold indicate the putative base-pairing region of *qrr*4 and are reverse-complement; nucleotides in box indicate the remaining sequence of *qrr*4) | Construction of anti-*qrr*4-pBAD33 plasmid |
| 5′ UTR *hapR* | TCTAGACTTTTAAAGCAATTAATAAAATAATCATTAGCGTTTACTAAAAAACGCAGATAACAACTCAAATGGCAAGGATTAATACTATGGACGCATCTATAGAGCAAGCTTGCGGCCGCATGGTGA | 126  (underlined nucleotides indicate restriction endonuclease sites of XbaⅠ and HindⅢ, respectively; nucleotides in red indicate the nucleotides used to design mutation in 5′ UTRm; nucleotides in grey indicate the start codon of *hapR*; nucleotides in box indicate the sequence in pET-28a-EGFP plasmid) | Construction of pET-5′ UTR *hapR*-EGFP plasmid |
| 5′ UTRm *hapR* | TCTAGACTTTTAAAGCAATTAATAAAATAATCATTAGCGTTTACTAAAAAACGCAGATAACAACTCAAATCCGAAGGATTAATACTATGGACGCATCTATAGAGCAAGCTTGCGGCCGCATGGTGA | 126  (underlined nucleotides indicate restriction endonuclease sites of XbaⅠ and HindⅢ, respectively; nucleotides in grey indicate the start codon of *hapR*; nucleotides in red indicate the three-nucleotide mutation; nucleotides in box indicate the sequence in pET-28a-EGFP plasmid) | Construction of pET-5′ UTRm *hapR*-EGFP plasmid |
| 5′ UTR *tssB*2 | TCTAGAGATATTTCTAATGTGTATTATCAATCAGATATTTACACAACTATTTCATTGACAACGACTGGCATAGGTATTTGGAGACGTTATATGTCTAAAGAAGGACAAGCTTGCGGCCGCATGGTGA | 127  (underlined nucleotides indicate restriction endonuclease sites of XbaⅠ and HindⅢ, respectively; nucleotides in grey indicate the start codon of *hapR*; nucleotides in box indicate the sequence in pET-28a-EGFP plasmid) | Construction of pET-5′ UTR *tssB*2-EGFP plasmid |
| 5′ UTR *tssD*2_a | TCTAGAGAATTCGCGCTGAGCGCACTTACATCACGAACGTTTTATCGAATGGCAAAGGTTGTCTTAACACGTTGCCTGATAAACAACCAGTTAGAAAGGAATGAGCAATGCCAACTCCATGTCAAGCTTGCGGCCGCATGGTGA | 144  (underlined nucleotides indicate restriction endonuclease sites of XbaⅠ and HindⅢ, respectively; nucleotides in grey indicate the start codon of *hapR*; nucleotides in box indicate the sequence in pET-28a-EGFP plasmid) | Construction of pET-5′ UTR *tssD*2_a-EGFP plasmid |

**References:**

1. Lu X, Liang W, Wang Y, et al. Identification of genetic bases of vibrio fluvialis species-specific biochemical pathways and potential virulence factors by comparative genomic analysis. Applied and environmental microbiology. 2014 Mar;80(6):2029-37.

2. Wang Y, Wang H, Liang W, et al. Quorum sensing regulatory cascades control Vibrio fluvialis pathogenesis. Journal of bacteriology. 2013 Aug;195(16):3583-9.
